# Study of Biological Effects Induced in Solid Tumors by Shortened-Duration Thermal Ablation Using High-Intensity Focused Ultrasound

**DOI:** 10.3390/cancers16162846

**Published:** 2024-08-14

**Authors:** Patrycja Maria Kaplińska-Kłosiewicz, Łukasz Fura, Tamara Kujawska, Kryspin Andrzejewski, Katarzyna Kaczyńska, Damian Strzemecki, Mikołaj Sulejczak, Stanisław J. Chrapusta, Matylda Macias, Dorota Sulejczak

**Affiliations:** 1 Department of Experimental Pharmacology, Mossakowski Medical Research Institute, Polish Academy of Sciences, Pawinskiego 5 St., 02-106 Warsaw, Poland; pkaplinska@imdik.pan.pl (P.M.K.-K.); sjchrapusta@imdik.pan.pl (S.J.C.); 2 Department of Ultrasound, Institute of Fundamental Technological Research, Polish Academy of Sciences, Pawinskiego 5b St., 02-106 Warsaw, Poland; lfura@ippt.pan.pl (Ł.F.); tkujaw@ippt.pan.pl (T.K.); 3 Department of Respiration Physiology, Mossakowski Medical Research Institute, Polish Academy of Sciences, Pawinskiego 5 St., 02-106 Warsaw, Poland; kandrzejewski@imdik.pan.pl (K.A.); kkaczynska@imdik.pan.pl (K.K.); 4 Department of Animal Physiology, Faculty of Biology, University of Warsaw, I. Miecznikowa 1 St., 02-096 Warsaw, Poland; m.sulejczak2@student.uw.edu.pl; 5 Laboratory of Molecular and Cellular Neurobiology, International Institute of Molecular and Cell Biology, Ks. Trojdena 4 St., 02-109 Warsaw, Poland; mmacias@iimcb.gov.pl

**Keywords:** HIFU thermal ablation, breast cancer model, treatment plan, morphology, histology, ultrastructure, immune response, cell death, apoptosis, necrosis

## Abstract

**Simple Summary:**

Breast cancer is one of the most dangerous cancers affecting women, so animal models of breast cancer have been created, new drugs are constantly being sought, and new procedures are being developed to destroy tumor cells. As to this last aspect, the use of high-intensity focused ultrasound is promising. It is a non-invasive technique, but its availability is significantly limited due to such devices’ costliness and lack of versatility. Therefore, this project aimed to construct a low-cost, compact HIFU for precise destruction of cancer cells in the region of interest and to test the efficacy of the shortened-duration ablation procedure in a rat model of implantable breast cancer. Our research is preclinical and may also be applicable in veterinary medicine.

**Abstract:**

The HIFU ablation technique is limited by the long duration of the procedure, which results from the large difference between the size of the HIFU beam’s focus and the tumor size. Ablation of large tumors requires treating them with a sequence of single HIFU beams, with a specific time interval in-between. The aim of this study was to evaluate the biological effects induced in a malignant solid tumor of the rat mammary gland, implanted in adult Wistar rats, during HIFU treatment according to a new ablation plan which allowed researchers to significantly shorten the duration of the procedure. We used a custom, automated, ultrasound imaging-guided HIFU ablation device. Tumors with a 1 mm thickness margin of healthy tissue were subjected to HIFU. Three days later, the animals were sacrificed, and the HIFU-treated tissues were harvested. The biological effects were studied, employing morphological, histological, immunohistochemical, and ultrastructural techniques. Massive cell death, hemorrhages, tissue loss, influx of immune cells, and induction of pro-inflammatory cytokines were observed in the HIFU-treated tumors. No damage to healthy tissues was observed in the area surrounding the safety margin. These results confirmed the efficacy of the proposed shortened duration of the HIFU ablation procedure and its potential for the treatment of solid tumors.

## 1. Introduction

Breast cancer is the most common disease and the leading cause of death in women [1,2]. It accounts for about 7–10% of all types of malignant neoplasms, and usually occurs in women aged 40–60 years. Worryingly, the incidence of breast cancer continues to rise [3,4]. Radical mastectomy (with or without excision of the pectoral muscle(s)) has long been the standard treatment for breast cancer. Currently, the standard of care for patients with operable breast cancer is breast-conserving surgery combined with radiotherapy [5].

Over the past decade, a number of innovative, potent, minimally invasive treatments based on ablation induction have been developed for breast cancer. They are an alternative to surgery and involve the induction of extreme hypo- or hyperthermia, which leads to cell damage and tissue loss [6]. The most intensively developed non-surgical ablation techniques have been shown to have a prognosis in terms of life expectancy for breast cancer patients similar to those of the standard treatment techniques. They are an attractive alternative for patients with small tumors of selected types of breast cancer in the early stages, as well as for local treatment in case of recurrence [7]. These techniques include cryoablation and thermoablation, such as laser ablation, radiofrequency ablation, microwave ablation, and high-intensity focused ultrasound (HIFU)-induced ablation. The last in the list is currently one of the most attractive techniques for the treatment of primary breast tumors [8,9]. It is also increasingly used as the therapy for other soft-tissue tumors, as well as in experimental and preclinical studies [10,11].

Recent developments in the use of HIFU thermoablation for the treatment of primary breast tumors are listed in a review publication [9]. The HIFU ablation technique is a percutaneous procedure mostly performed using an extracorporeal HIFU ablation device under the guidance of either magnetic resonance imaging (MRI) or ultrasound imaging (USI). The basic principle of the HIFU technique is the thermal destruction of the tumor—located deep under the skin—without damaging the surrounding healthy tissues. The tumor is destroyed by the induction of coagulative necrosis [12] in multiple small ellipsoidal volumes within it (in the focal volume of the HIFU beam) due to the absorption of the energy of ultrasound waves penetrating through the skin and focused inside the tumor, as well as due to inertial cavitation.

The main advantages of the HIFU technique are non-invasiveness (no surgery), lack of harmful ionization, the possibility of multiple repetitions of the procedure, and minimal side effects compared to conventional treatment methods (radiotherapy or chemotherapy, or surgery). Due to the non-invasive nature of the procedure, HIFU thermal ablation therapy causes fewer side effects than other minimally invasive ablation methods used in clinical practice, such as cryoablation, ablation induced by percutaneous alcohol injection, and thermoablation induced by radiofrequency or microwaves [9].

The breast is well suited for thermoablation of deep-seated solid tumors using the HIFU technique, because it is easily immobilized (thus eliminating unwanted breathing-related movements). It also has a large surface area (acoustic window on the skin) through which ultrasound waves can penetrate deep into the underlying tissues, concentrating their energy in the beam’s focus, which is navigated to the treated volume. As the size of tumors subjected to HIFU ablation is much larger than the ellipsoidal focal volume of the HIFU beam, to ensure the destruction of the tumor it is necessary to scan the entire tumor volume with the focal volume of the HIFU beam. This is usually performed by moving the focus of the HIFU beam with a mechanical or electronic precise positioning system. The margin between completely destroyed cells at the periphery of the tumor and healthy tissue is less than 50 μm thick [8,9].

Commercial HIFU devices guided either by ultrasound imaging (USIgHIFU) or magnetic resonance imaging (MRIgHIFU) have been used in clinical practice to ablate solid tumors of various organs [13,14,15,16,17,18,19,20,21]. The main advantage of USIgHIFU ablation systems over those associated with MRIgHIFU is the ability to monitor the procedure in real time, as well as lower equipment and operating costs. However, there are no commercially available compact USIgHIFU devices available for preclinical studies which are equipped with a computer system that can be used to automatically control the acoustic properties of each single HIFU beam, its movement trajectory, and the time intervals between consecutive sonications. Therefore, such a system could become an important research tool for the development of effective and safe treatments of solid tumors.

For this purpose, we have designed, developed, and built an automated USIgHIFU ablation device for preclinical studies in small animals [22]. It is a low-cost, compact, automated HIFU ablation system, whereas commercially available devices (e.g., the HIFUPlex Plus 1000 developed by Verasonics and VIFU 2000 developed by Alpinion Medical Systems) are very expensive. Our device was designed and built for preclinical research and is suitable not only for thermoablation of solid tumors in small animals, but also for testing the targeted delivery of new anticancer drugs released under ultrasonic hyperthermia. Our device includes a HIFU transducer integrated with an ultrasound imaging probe linked to a diagnostic ultrasound scanner, an electronic power system, a computer-controlled mechanical system for precise positioning of the HIFU beam’s focus in the focal plane, and a notebook with custom software (developed in-house based on Java v8.) for planning and performing the ablation procedure. The ultrasound imaging probe is used to guide the focus of the HIFU beam to the target.

One of the reasons limiting the wider use of the HIFU ablation technique is the long treatment time resulting from, among other causes, the large difference between the size of the heating beam’s focus and the size of the tumor, as well as the long time intervals between single sonications. The treatment of larger tumors requires sonication of their volume with a sequence of single HIFU beams, the focus of which is moved in the focal plane along a specific trajectory with a specific time interval between sonications. During the procedure, to avoid an undesirable increase in the temperature of healthy tissues surrounding the tumor, the average acoustic power of each pulsed HIFU beam and exposure time to it, as well as the time interval between consecutive sonications, should be selected in such a way as to cover the entire volume of the tumor with necrosis as quickly as possible. This reduces treatment costs and allows for more ablations to be performed. 

In this study, we developed and tested a new tumor treatment strategy that dramatically reduced the duration of the HIFU treatment procedure. The duration of the ablation procedure in our studies was largely determined by the short time available, during which the animal was kept motionless after the administration of a specific dose of anesthesia. The proposed procedure was designed based on our earlier results [23,24]. An additional advantage of our ablation procedure is its automatized character. The influence of the acoustic properties of the HIFU beam used (acoustic power, duty factor), its trajectory, the time intervals between consecutive sonications, and the exposure time on the efficacy of necrosis induction was evaluated within the entire volume of the treated tumors. The acoustic parameters of the HIFU beam, as well as the location and size of the necrotic lesions induced by it inside the tissue, have been determined in our previous studies [23,24]. Necrotic cell death is associated with the induction of an inflammatory response. Thus, another goal of this study was to determine whether HIFU treatment causes an influx of immune cells into the tumor area and the expression of pro-inflammatory cytokines: interleukin-1α (IL-1α), interleukin-1β (IL-1β), interferon γ (IFN-γ), and tumor necrosis factor α (TNF-α). The efficacy of the HIFU ablation was evaluated at both the cellular and sub-cellular level using a variety of light, fluorescence, and electron microscopy (EM) techniques.

## 2. Materials and Methods

### 2.1. Device Configuration

**Figure 1:**
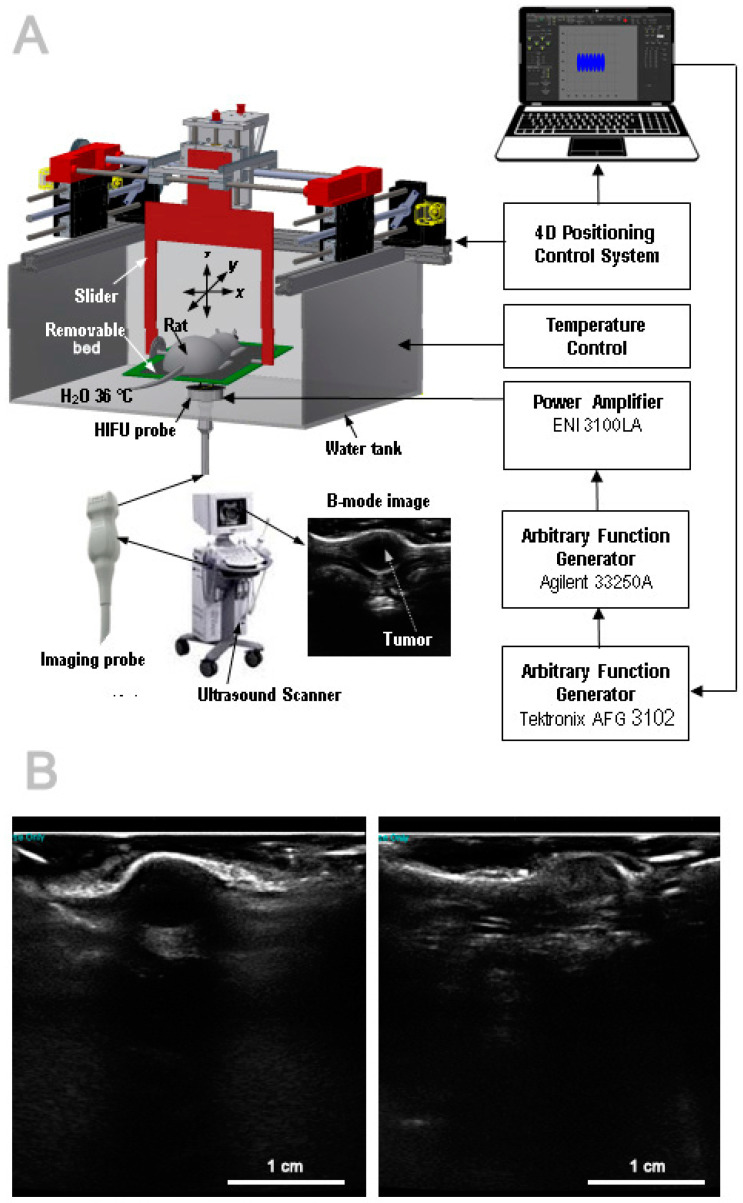
Block diagram of the equipment used for the ablation of tumors in small animals by HIFU beams (**A**). Representative ultrasound images of axial tumor cross-sections taken just before (**left**) and just after (**right**) sonication (**B**).

A block-diagram of the equipment used for tumor ablation is presented in Figure 1A.

The USIgHIFU ablation system consisted of an H102 bowl-shaped heating transducer (Sonic Concepts Inc., Bothell, WA, USA) coaxially integrated with a Zonare P10-4 imaging probe mounted in its 20-mm central opening and connected to a Zonare ultrasound scanner (Zonare Medical Systems Inc., Mountain View, CA, USA), which was used to guide the focus of the HIFU beam to the target in real time. The diameter of the HIFU transducer was 64 mm, the focal length was 62.6 mm, and the operating frequency was 3.21 MHz. The imaging probe operated at a frequency of 8 MHz. The integrated heating and imaging probe was mounted in the bottom of a bath filled with degassed water. The constant temperature of the water in the bath (36 °C) was maintained by means of a thermostat used with an Aquael 100 electric heater (Aquael Ltd., Warsaw, Poland) and an aquarium pump submerged in the water. The HIFU transducer was driven by electrical sinusoidal pulses of selected amplitude, duration, and duty-factor by means of an electronic system consisting of an Agilent 33250A function generator (Colorado Springs, CO, USA) and an ENI 3100LA power amplifier (Rochester, NY, USA). To ensure that the same number of pulses were delivered during a single exposure to a HIFU beam, the AFG 3102 function generator (Tektronix Inc., Beaverton, OR, USA) was applied. The output signal from the amplifier excited the HIFU transducer, which produced a pulsed beam with an average acoustic power of 108 W, pulse duration of 0.3 s, and duty factor of 0.6 (0.3 s ON + 0.2 s OFF). The length and diameter of the ellipsoidal necrotic lesion formed inside the tissue (at the focus of the HIFU beam) as a result of its single 3 s exposure (a train of 10 pulses) to such a beam were approximately 7 mm and 1.7 mm, respectively. These values were determined in our previous studies [23,24,25]. 

Above the integrated heating and imaging probe was a frame (with a removable treatment bed for placing the animal) connected by a slider to a mechanical system which enabled the precise positioning of the frame within the xyz coordinate system at a specific inclination angle (0° in the present study). The precise positioning system was placed on the top of the water bath. The frame, the treatment bed, and the abdomen of the animal were immersed in the water filling a 30 × 30 × 15 cm bath; the water had a dual role as a matching medium between the HIFU transducer and the animal’s body and as a cooling medium used to prevent skin burns. The water layer between the center of the HIFU transducer and the animal’s abdomen was approximately 55 mm in height. After programming the trajectory of the treatment bed and the time intervals between sonications, the system was set to automatically carry out multiple exposures of the tumor to the HIFU beam. 

The treatment bed had a round opening through which the HIFU beam penetrated the rat’s body. A thin ultrasound-transparent foil was stretched over this hole to prevent the abdomen from falling out. Through this opening (located coaxially with the integrated heating and imaging probe) the energy of each HIFU beam penetrated the rat’s body and was concentrated in a small volume inside the tumor (at the heating beam’s focus). This caused an increase in its temperature, which led to its coagulative necrosis. Due to the coaxiality of the ultrasound imaging probe and the HIFU transducer, the focus of the heating beam in the original position was always located on the central axis of the ultrasound image at a known depth determined by the focal length of the HIFU transducer used (as specified by the manufacturer) and the depth of the water layer (approximately 55 mm) between the transducer and the rat body.

### 2.2. Rat Model of Implanted Breast Cancer

All experimental procedures and animal treatments were approved by the II Local Ethics Committee for Experiments on Animals at the Warsaw University of Life Sciences (permit No. WAW2/042/2019). The experimental protocols followed the guidelines published in European Directive 2010/63/EU on the protection of animals used for research purposes.

Twenty-four young adult male Wistar rats weighting 250–260 g were each given a single subcutaneous injection of 2 × 10^6^ mammary adenocarcinoma (13762 MAT B III [ATCC CRL-1666TM], ATCC, Manassas, VA, USA) cells in a 200 µL fluid medium above the right hip under short isoflurane anesthesia. This location permits the tumor to grow freely without distressing the animal. The tumors were then allowed to develop for about one week until they reached a diameter of 6 mm. The rats were then divided into two groups: HIFU-treated (*n* = 12) and control (*n* = 12). The control-group rats were scanned with diagnostic ultrasound for the tumor visualization exactly like the other group, but were not treated with HIFU. Diagnostic ultrasound images (B-mode) of axial tumor cross-sections from HIFU-treated rats were acquired just before and just after sonication. The tumor areas on the B-mode images were identified and analyzed in terms of the tumors’ location and size. Example B-mode images of the central axial tumor cross-section before and after sonication are shown in Figure 1B. 

### 2.3. Ablation Planning

Tumors undergoing ablation had the shape of a ball with a diameter of approximately 6 mm, and their centers were approximately 8 mm below the skin surface. The focus of the HIFU beam was guided to the tumor’s center by adjusting the position of the treatment bed under the guidance of ultrasound imaging. Taking into account the geometry of the HIFU transducer (focal length 62.7 mm) and the depth of the water layer between it and the animal’s skin, the beam’s focus was always at a depth of approximately 8 mm under the skin. As mentioned above, the heat source was located approximately 55 mm from the skin. The water temperature in the water bath, regulated by a thermostat, was 36 °C.

The tissue volume planned for ablation was defined as an 8 mm diameter, 7 mm long cylinder (encompassing the entire 6 mm tumor with a 1 mm safety margin of healthy tissue around it), with the center located about 8 mm below the skin’s surface. This had been determined based on our previous studies. The size of the treated tumor was determined just before the start of the ablation procedure, based on measurements of its cross-sections on ultrasound images and an electronic caliper. The total tumor volume without a margin was about 0.113 cm^3^. 

The size of the tumor planned for ablation (with the safety margin around it) was many times larger than the size of the HIFU beam’s focus (an ellipsoid with a diameter of 1.7 mm and a length of 7 mm). To ensure complete thermal destruction of the tumor, it was necessary to sonicate it many times with a single beam, the focus of which was moved in the focal plane along a specific trajectory, while maintaining a specific time interval between sonications.

The development of the treatment plan involved programming the trajectory of tumor sonications by the HIFU beam in the focal plane (xy) perpendicular to the ultrasound imaging plane (xz), as well as the time interval between sonications, so as to induce necrosis of the entire tumor volume in the shortest possible time.

**Figure 2:**
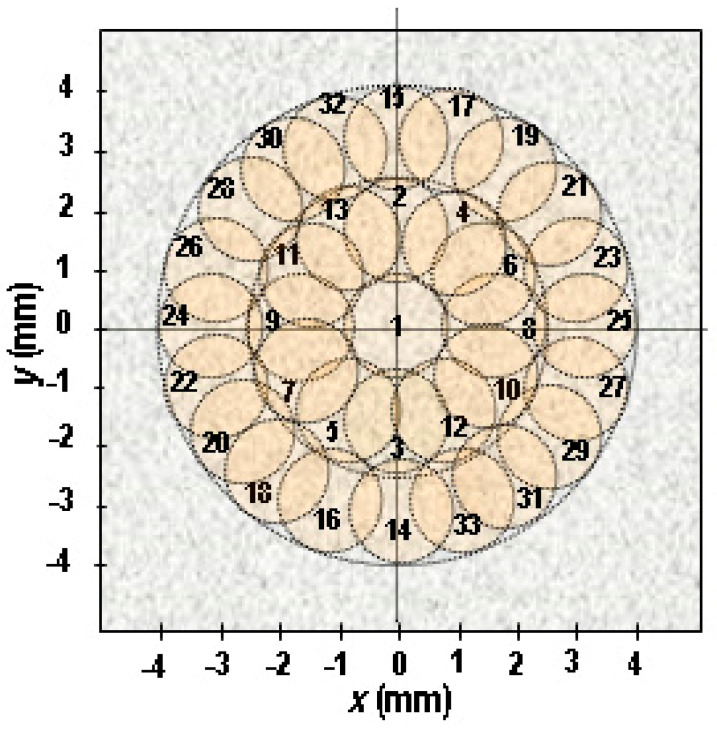
Distribution and sequence of the exposures of a 6 mm diameter tumor (plus a 1 mm margin around it) to a single 3.21 MHz HIFU beam moved along the planned trajectory. Radial sections of the planned ellipsoidal necrotic lesions, which are intended to summarily form an 8 mm diameter cylindrical necrotic area of damage encompassing the entire tumor, are shown.

The distribution and sequence of sonications and the cross-sections of the necrotic lesions induced by them in order to create a cylindrical necrotic area of damage with a diameter of 8 mm and a length of 7 mm are shown in Figure 2.

Tumor ablation was performed automatically after programming the trajectory of the HIFU beam and the time interval between individual sonications using the device’s computer control system. 

In previous studies [24] assuming the same ablation plan, the exposure time of the examined tissue to a single HIFU beam was 3 s. The time interval between consecutive sonications, equal to 120 s, resulted from the temperature relaxation time of the tissue examined. Then the duration of the ablation procedure, which was the sum of the times of 33 single (3 s) exposures and 32 timed breaks (120 s) between them, was 65.5 min. In this in vivo study, taking into account blood perfusion in living tissues, the same ablation plan and exposure time to a single beam (3 s) was used, but a shortened time interval between sonications (20 s) was adopted. In this case, the duration of the entire ablation procedure was reduced more than 5-fold to just over 12 min. Our recent study [25] has shown that shortening the time interval between subsequent sonications does not significantly increase the size of the resulting lesion if the other input parameters remain the same.

To avoid overheating the healthy tissues surrounding the tumor, the HIFU beam trajectory was selected to space consecutive ablations as far apart as possible (see Figure 2), which thereby reduced the impact of the residual heat remaining at the site of the previous ablation on the size of the necrotic lesion at the time of the next sonication. The higher the initial tissue temperature, the greater the size of the necrotic lesion resulting from sonication by a HIFU beam with the same sonication parameters.

### 2.4. HIFU-Ablation Procedure

The animal was placed prone on the treatment bed so that the tumor was in the center of the round opening cut in the bed. Under the guidance of ultrasound imaging, the focus of the HIFU beam was targeted to the center of the tumor using a precise positioning system. Thanks to the ability to move the treatment bed with the animal in both directions, i.e., parallel and perpendicular to the imaging plane, it was possible to determine the location and volume of tissue intended for ablation based on ultrasound images, as well as to program the tumor the trajectory of the HIFU beam and the time interval between sonications.

The movement of the treatment bed was computer-controlled using custom software (developed in-house based on Java v8.), and its input parameters were determined from the location and size of the tumor, as measured on cross-sectional ultrasound images. The formation of each necrotic lesion during a single exposure took 3 s. The time intervals between consecutive sonications were 20 s. Taken together, these modifications made it possible to reduce the total duration of the ablation procedure by more than 5 times. The total procedure duration was approximately 12 min per rat.

To ensure that the entire tumor was covered with necrosis, the diameter of the cylindrical tissue volume to be ablated was about 2 mm larger than the tumor diameter and was centered on its axis at a known depth below the rat skin. 

Criteria for inclusion of a rat in the study included the following: pathologically proven breast cancer, single palpable tumor of 6 mm in diameter, center of the pathological lesion visualized by ultrasonic imaging, and the tumor being located at least 8 mm below the skin surface. 

The procedure was conducted as follows. First, the rat was given general anesthesia (80 mg/kg ketamine and 7 mg/kg xylazine, i.m.). The skin area around the tumor was then shaved with an electric shaver and smoothed with a depilatory cream. Once the cream was washed off, the animal was placed prone on the surface of a removable treatment bed with a 50 mm diameter opening sealed with polyethylene foil to prevent the abdomen from falling into the hole, and immobilized. The center of the tumor was situated in the center of the opening. Before the procedure, ultrasound images of the tumor’s axial sections were obtained. The treatment bed with the animal was placed on the frame connected to the slider of the mechanical system, which was used for precise positioning, and immersed in water, which served as a coupling and cooling medium. Ultrasonic beams penetrated the skin of the animal, and their energy was focused within the targeted volume within the tumor. Ultrasonic imaging was used to guide the focus of the HIFU beam to the target and to plan and perform the treatment. In order to perform the ablation procedure, automatic treatment of the tumor with the focus of the HIFU beam was started according to the programmed trajectory of its movement in the focal plane. The entire tumor was scanned, along with a 1 mm margin of the surrounding tissues. 

Ultrasound images taken immediately after ablation were compared with those taken immediately before treatment to determine changes in the degree of echogenicity (grayscale), which defines the extent of thermal damage to the tumor after HIFU treatment. The appearance of a hyperechoic area on the ultrasound image indicated the efficacy of the ablation.

The vital signs of the rats under study were stable. Following HIFU ablation, rats were observed for potential post-treatment side effects, such as skin burns, local pain or discomfort, edema, infection, and fever. After the sonication, the animals received appropriate doses of analgesic and anti-inflammatory drugs for three consecutive days.

### 2.5. Bioeffects Detection

The effects of HIFU treatment were analyzed 3 days after sonication. This time-point was chosen based on the results of preliminary studies in which animals were examined 24, 48, and 72 h after treatment. The greatest changes were observed 3 days post-treatment. For ethical reasons, a longer follow-up time could not be used, because by that time the tumor burden in the respective control animals had reached a scale that required the rats to be sacrificed. Preliminary studies had shown that if the tumor was not-quite-successfully treated with HIFU, it regrew during 3 post-sonication days from the cells left behind and reached a size of approximately 1.0–1.5 cm. 

The animals were sacrificed under deep anesthesia (Morbital, 150 mg/kg) and the tumors, along with the surrounding healthy-tissue margins, were removed and fixed in 4% formaldehyde solution in a 0.1 M phosphate buffer at pH 7.4 for 5 days for morphological and immunohistochemical studies (light and fluorescent microscopy) or in formaldehyde (2% *w*/*v*) and glutaraldehyde (2.5% *w*/*v*) in 0.1 M cacodylate buffer pH 7.4 for 20 h for ultrastructural studies. The fixed material was next processed according to standard procedures for microscopic examination, as briefly described below.

Specimens for EM were postfixed in 1% (*w*/*v*) OsO_4_ solution in deionized water for 30 min, dehydrated in an ethanol gradient, and finally encased in epoxy resin Epon 812 and sectioned with a microtome. The sections were analyzed in a transmission electron microscope (JEM-1200EX, JEOL, Tokyo, Japan). 

The fixed material for light and fluorescence microscopy was then cryopreserved by immersion in 10, 20, and 30% (*w*/*v*) sucrose solutions in PBS (for 24 h, 3 days, and 6 days, respectively). The samples were frozen on dry ice and cut into 20 µm-thick sections using a CM 1850 UV cryostat (Leica, Wetzlar, Germany). Half of each section was used for morphological studies using routine hematoxylin and eosin (H&E) staining, and the other half was used for immunohistochemistry (IHC). The stained sections were analyzed using a Nikon Eclipse Ni-U light microscope (Tokyo, Japan) equipped with a CCD camera (DS-L1, Tokyo, Japan) and image analysis system.

Single and double IHC procedures were performed according to routine protocols. To diminish background staining, the sections were preincubated with 3% normal goat serum solution in PBS supplemented with 0.2% Triton X-100 (PBS+T) for 20 min at room temperature. Then, the sections were incubated for 1 h at 37 °C with PBS+T containing 1% normal goat serum and one or two of the following primary antibodies (Abs): (1) murine monoclonal Ab against vascular endothelial growth factor (VEGF) (Santa Cruz Biotechnology, Santa Cruz, CA, USA, dil. 1:400); (2) rabbit polyclonal Ab against IL 1-β (Invitrogen-Molecular Probes, Eugene, OR, USA, dil. 1:400); (3) murine monoclonal Ab against IL 1-α (Santa Cruz Biotechnology, CA, USA, dil. 1:400); (4) murine monoclonal Ab against TNF-α (Santa Cruz Biotechnology, CA, USA, dil. 1:400); (5) murine monoclonal Ab against macrophages (Santa Cruz Biotechnology, CA, USA, dil. 1:400); (6) murine monoclonal Ab against lymphocytes CD8 (DAKO, Santa Clara, CA, USA, dil. 1:400), and (7) murine monoclonal Ab against INF-γ (Santa Cruz Biotechnology, CA, USA, dil. 1:400). Next, the sections were washed with PBS+T (3 × 5 min) and subsequently incubated for 1 h at 37 °C with the respective secondary Abs: goat anti-mouse Ab conjugated with Alexa Fluor 594 (Invitrogen-Molecular Probes, Eugene, OR, USA, dil. 1:100); and goat anti-rabbit Ab conjugated with Alexa Fluor 488 (Invitrogen-Molecular Probes, Eugene, OR, USA, dil. 1:100). Finally, the sections were rinsed with PBS+T (3 × 5 min) and cell nuclei were counterstained by 10 min incubation with bisbenzimide (Hoechst 33342, Thermo Fisher Scientific Inc., Waltham, MA, USA; Hoechst staining) solution in PBS (5 ng/mL). Next, the dye solution was removed and the sections were covered with Vectashield mounting medium for fluorescence microscopy (Vector Labs Inc., Burlingame, CA, USA) and analyzed using an Eclipse Ni-U light fluorescent microscope (Nikon, Tokyo, Japan) equipped with appropriate filters, a DS-L1 CCD camera (Nikon, Japan), and a computerized image analysis system. Specificity of the immunostainings was verified by performing control labeling procedures, with primary antibodies omitted in the corresponding incubation mixtures. No staining was detected in the respective control slices.

### 2.6. Visualization, Quantification, and Statistical Analysis

The expression levels of pro-inflammatory cytokines and of VEGF were determined using immunohistochemical labelling techniques. Investigated sections were captured and analyzed. Acquisition was performed with a magnification of 40×, using high resolution. Computerized image processing methods were used to assess the level of immunoreactivity. The image processing software ImageJ 1.43u (Developer: Wayne Rasband, National Institutes of Health, Stapleton, NY, USA) was used for analysis. Acquired sections were converted to greyscale and calibrated to 8-bit scale, which allows the detection of 256 levels of staining intensity measured as an optical density, and plot profiles were generated. The heights of the respective surface plot profiles represent the intensity levels of the immunoreactive signal.

To determine the effect of the HIFU, the number of leukocytes infiltrating the tumor area was assessed. The number of blood vessels and extravasations in the tumor area were also determined. Measurements were taken from two randomly selected microscopic fields of view within the tumor section for each animal in the HIFU-treated group and the control HIFU-untreated group. Results are presented in graphs as mean number of cells, blood vessels, or extravasations (respectively), ±SD. Data were statistically analyzed using the Mann–Whitney test for independent variables. In all cases, *p* < 0.05 was considered significant. All statistical analyses were performed using STATISTICA v. 12 software (StatSoft Inc., Tulsa, OK, USA).

## 3. Results

The aim of the research was to assess the feasibility of utilizing the developed ablation plan, which significantly shortens the duration of therapy, to effectively target the destruction of cancer cells while minimizing collateral damage to the healthy tissues surrounding the tumor. For the evaluation, the techniques of histological examinations conducted at the tissue/cellular and sub-cellular level were used, which allowed for the assessment of the condition of the examined tumor cells and adjacent tissue(s).

### 3.1. Verification of the HIFU Therapy Efficacy Using Light Microscopy Imaging Techniques

**Figure 3:**
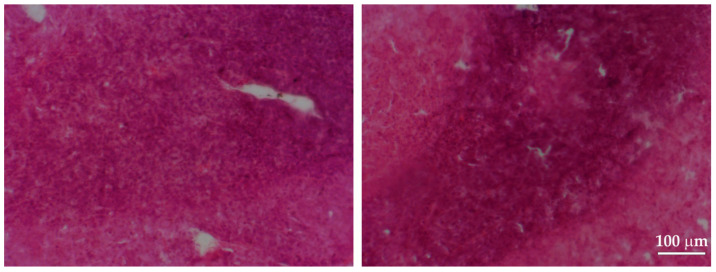
Representative microphotographs of HE-stained sections of a tumor from a control group of rats (HIFU-untreated). See a clearly visible dense structure of tumor with typical cell distribution.

**Figure 4:**
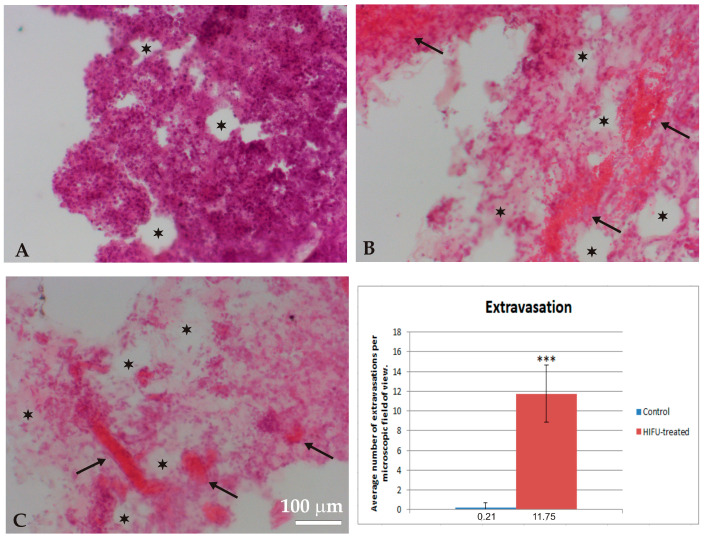
Microphotographs showing HE staining of HIFU-treated tumor 3 days post-sonication. Notice the leukocyte infiltration (**A**), hemorrhages and extravasation (**B**), and tissue atrophy, i.e., cell thinning and loss (**C**). Arrows indicate hemorrhages. Asterisks show areas with cell loss. The graph shows the average number of extravasations visible in the microscopic field of view. The data were tested using the Mann–Whitney test for independent groups, *** *p* < 0.001 versus control group.

The untreated tumors showed a compact structure and arrangement of tumor cells. No damage was observed (Figure 3). Only a few isolated extravasations were observed inside tumors from four of the control animals (1 extravasation per microscopic field of view, see graph in Figure 4). No extravasations were observed in tumors from the remaining control animals.

HIFU sonication induced marked changes in the structure and morphology of the tumor, leading to cell death and tumor destruction during the 3 days after tissue sonication with HIFU. The influx of immune system cells was observed in the sonication-affected region. A significant number of extravasations and micro-hemorrhages were demonstrated, as well as a clear loss of cells and damage to the tumor tissue. Figure 4 shows representative micrographs of various regions of HIFU-treated tumors, labeled with H&E and scored 3 days after sonication. In the cross-sections of the volumes treated with HIFU, areas of tumor lesions with distinct tissue defects and tearing, as well as massive inflows of leukocytes into the neoplastic volume, are visible (Figure 4A). Numerous hemorrhages within and around the tumor were also observed (Figure 4B), as well as tissue defects and areas with marked cell thinning or the complete loss of the cells (Figure 4C).

### 3.2. Verification of the HIFU Ablation Efficacy Using Electron Microscopy Imaging Techniques

**Figure 5:**
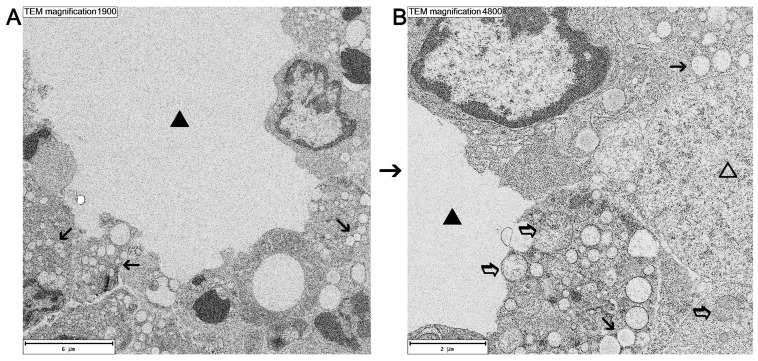
Electron microscope image of tumor, 3 days after ablation. (**A**) An area of tumor completely devoid of cells and filled with material from extravasated plasma (black triangle) is adjacent to degenerating cells with swollen organelles (black arrows). (**B**) Excerpt from the microphotograph shown in panel (**A**) at higher magnification. Degenerating cells show a significant number of vesicles in the cytoplasm (black arrows). On the right side—degenerating cell with a rarefied cytoplasm (transparent triangle). Transparent arrows indicate swollen mitochondria.

Electron microscopy studies confirmed the observations revealed by light microscopy techniques. They showed that the HIFU ablation procedure leads to massive damage and degeneration of tumor cells. Figure 5 illustrates a large tumor region devoid of cells, with clearly visible tissue swelling and extravasated plasma. Confirmation of this can be found on the obtained images as numerous extravasations, edema, and interstitial effusions observed in previous light microscopic examinations. The cells at the periphery of the extravasation show marked signs of degeneration. There are numerous vesicles within the cytoplasm and sites of damage to the organelles, with especially significant destruction of mitochondria. The degenerating cells also had altered cell nuclei.

**Figure 6:**
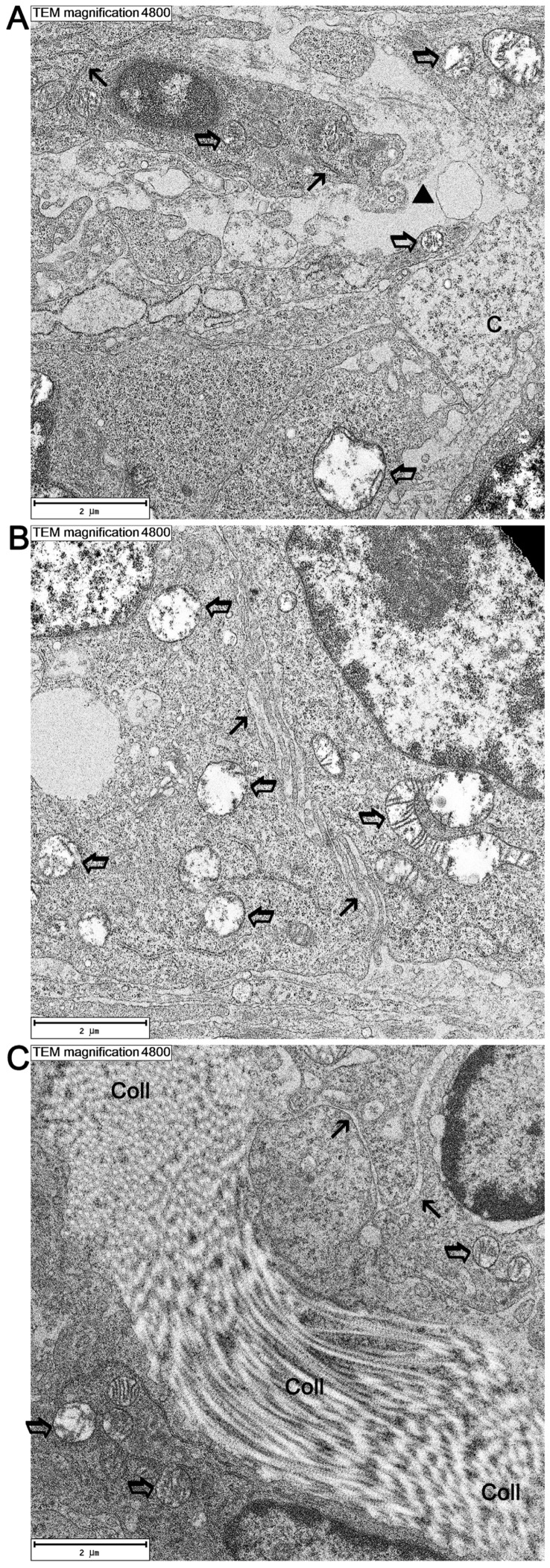
Ultrastructure of cells within tumor parenchyma, 3 days following HIFU treatment. (**A**) Degenerating cells seen against the stromal background, with extravasated plasma (black triangle) in swollen tissue. Note the swollen organelles inside the cells. C—necrotic cell with thinned cytoplasm. (**B**) Degenerating cells presenting reduced numbers of organelles, edema, and thinned cytoplasm. (**C**) Collagen fibers in various cutting planes (Coll) between cells, with features of degeneration, including pronounced swelling of the cytoplasm and cell organelles. Transparent arrows indicate swollen mitochondria. Black arrows show swollen endoplasmic reticulum cisternae.

Numerous swellings were also observed in the tumor parenchyma, in the vicinity of which there were cells at various stages of degeneration (Figure 6). Their cytoplasm was significantly thinned with a clear loss of cell organelles. A significant number of necrotic cells were observed at various stages of the necrotic process. Numerous remains of dead cells were also found, and an increase in the number of immune cells flowing into the area studied. A significant number of collagen fibers (from the dermis) were observed in the vicinity of the tumor (Figure 6C).

The EM results confirmed our earlier observations, indicating the effective destructive effect caused by thermo-ablation of the tumor using the HIFU technique. In addition, ablation experiments were performed in a limited, non-total tumor volume to assess the ability of the untreated part of the tumor to grow and reconstruct the tumor. This provided more information on the size of the applied margin, which is still safe and ensures the success of the treatment of the studied tumor, e.g., in a situation where the tumor grows in a “diffuse” manner, and it is possible to omit a small part of it during the ablation procedure. The results obtained indicated that leaving even a small tumor part uncovered with ablation resulted in tumor regrowth, which was observed as soon as 3 days after incomplete HIFU therapy.

### 3.3. Verification of HIFU Ablation Efficacy Using Fluorescent Microscopy Imaging Techniques and Immunofluorescent IHC Methods

**Figure 7:**
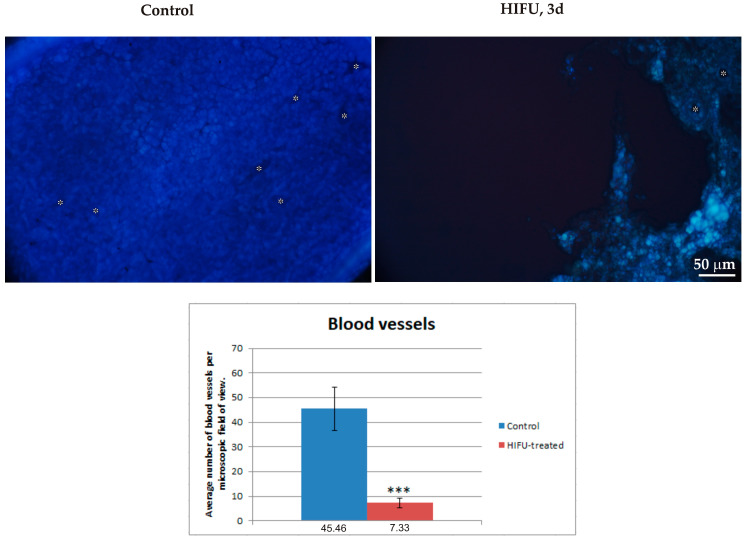
Cell nuclei of control and ablated tumors stained with bisbenzimide (Hoechst staining). Asterisks show the blood vessels. Graph shows the average number of blood vessels in the microscopic field of view. The data were tested using the Mann–Whitney test for independent groups, *** *p* < 0.001 versus control group.

Fluorescent labeling of tumor cell nuclei confirmed the morphological and EM findings presented above. Tumors harvested from control (i.e., not subjected to HIFU) showed a solid structure with densely arranged cells and numerous blood vessels. Three days after sonication, massive loss of tumor tissue was observed. The remaining tumor fragments showed marked thinning of cells and a small number of blood vessels (Figure 7). 

**Figure 8:**
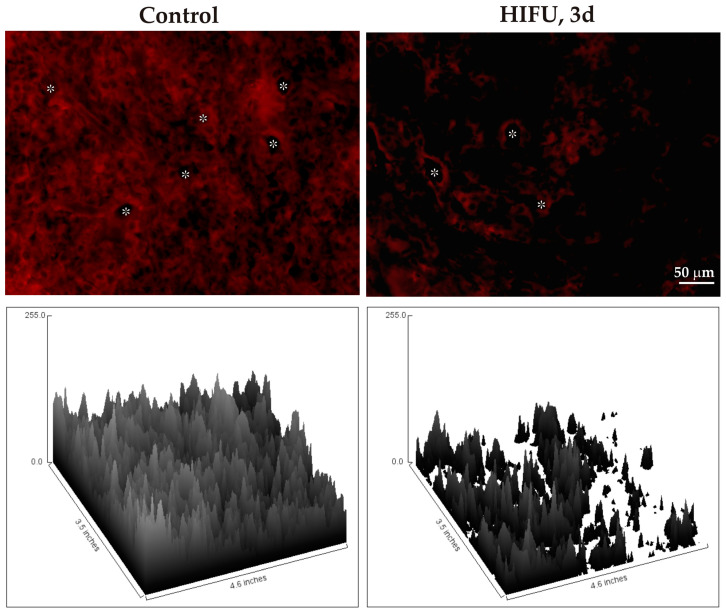
VEGF IHC (red) in the untreated (control) and HIFU-treated tumors (3 days post-treatment) (**upper panel**). Asterisks show the lumina of blood vessels. The intensity of immunostaining is shown as the heights of the relevant surface plot profile (**lower panel**).

IHC labeling of tumors ablated with HIFU showed a marked decrease in VEGF immunoreactivity, as compared to that in the control group, which clearly correlated with reduced number of blood vessels in the HIFU-treated tumor (Figure 8).

**Figure 9:**
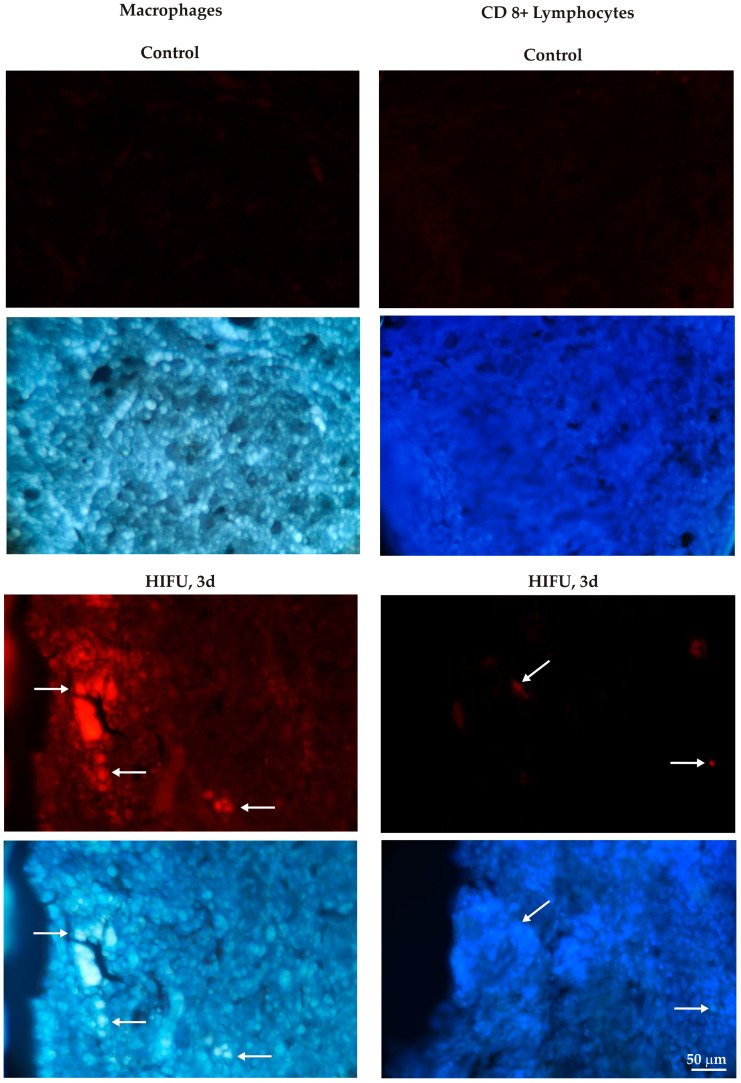
Control and HIFU-treated tumors immunolabeled for macrophages (red, (**left**) panel), and CD8+ lymphocytes (red, (**right**) panel). White arrows show immunopositive cells. Cell nuclei are stained with bisbenzimide (blue).

**Figure 10:**
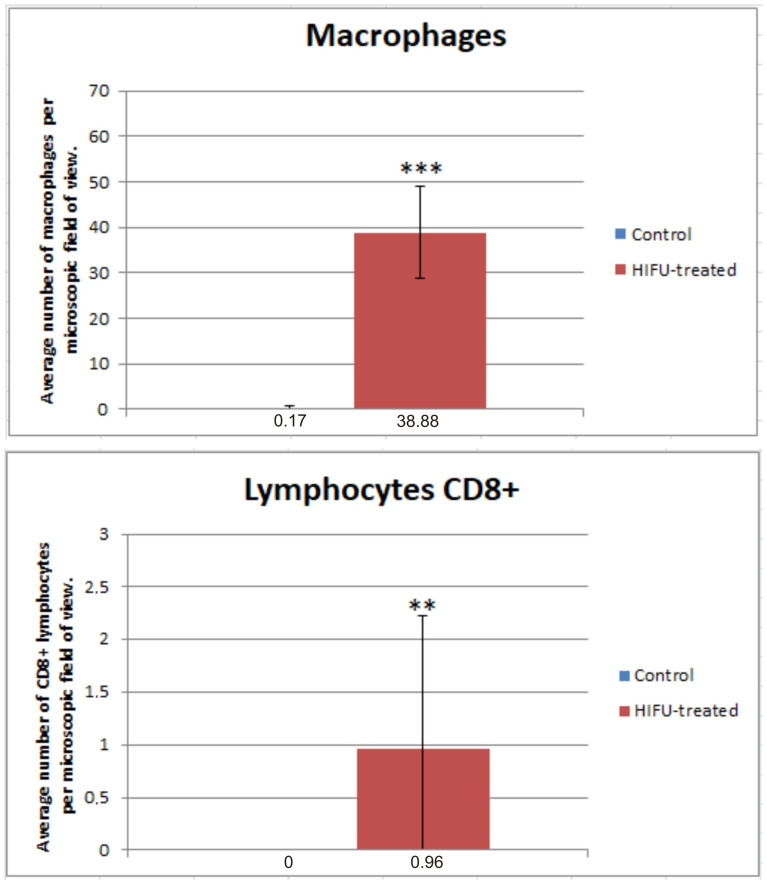
Graphs showing the average number of macrophages and CD 8+ lymphocytes visible within the microscopic fields of view. The data were analyzed using the Mann–Whitney test for independent groups, ** *p* < 0.01, *** *p* < 0.001 versus control group.

Three days after sonication, an influx of immune cells was observed in the remaining tumor fragments, and inflammation developed in the HIFU-treated tumor. The cells registered were mainly macrophages, whereas only single CD 8+ lymphocytes were found in the treated tumor. Single macrophages were occasionally seen in control tumors (Figure 9 and Figure 10).

Figure 10 shows the differences in the number of immune cells visible within the tumor areas between the control group and the HIHU-treated group.

**Figure 11:**
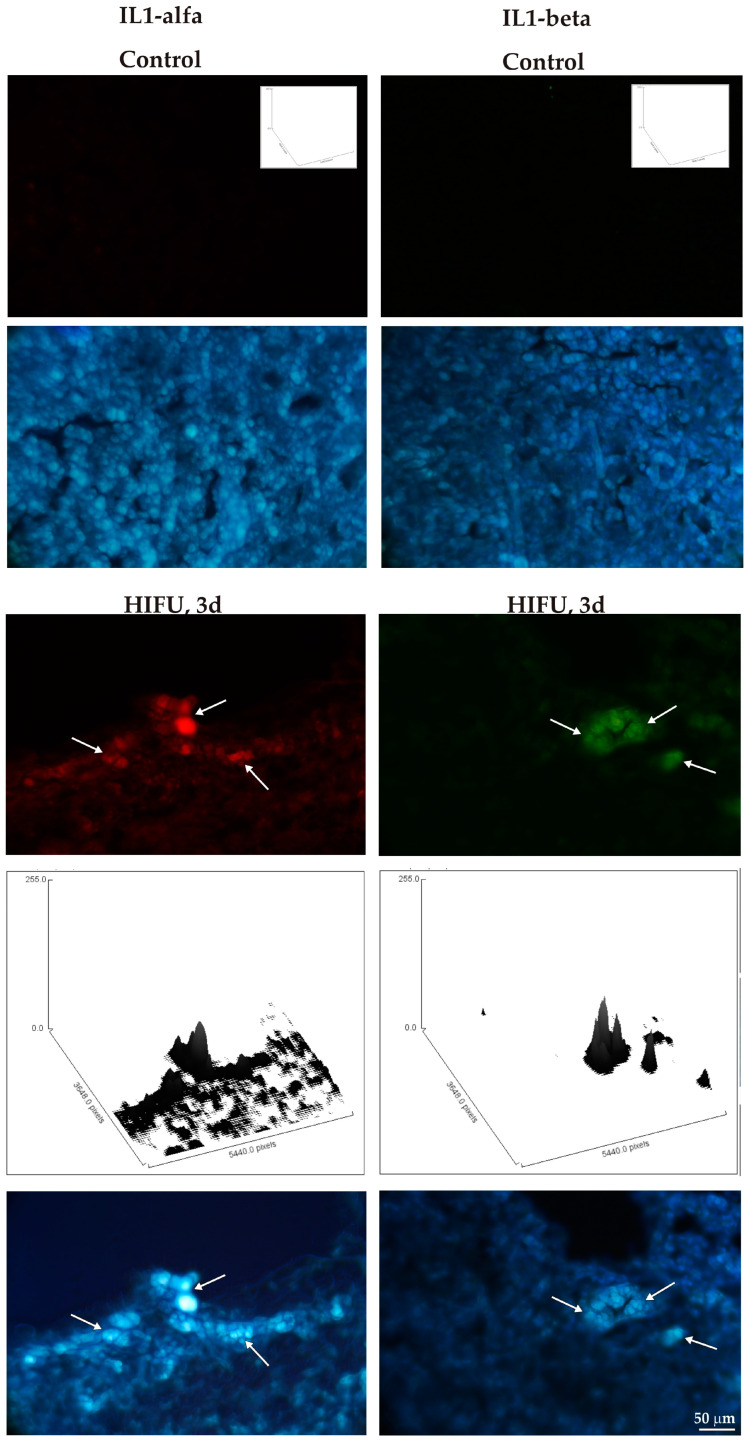
Immunolabeling for IL-1α (red, (**left**) panel) and IL-1β (green, (**right**) panel) in a HIFU-treated tumor (3 days post-sonication) as compared to an untreated control tumor. White arrows indicate immunopositive cells. Bisbenzimide was used for visualization of cell nuclei (blue). Histograms show the intensity of immunolabeling for IL-α and IL1-β per microscopic field of view. The intensity of immunoreactivity is presented as the height of the surface plot profiles.

**Figure 12:**
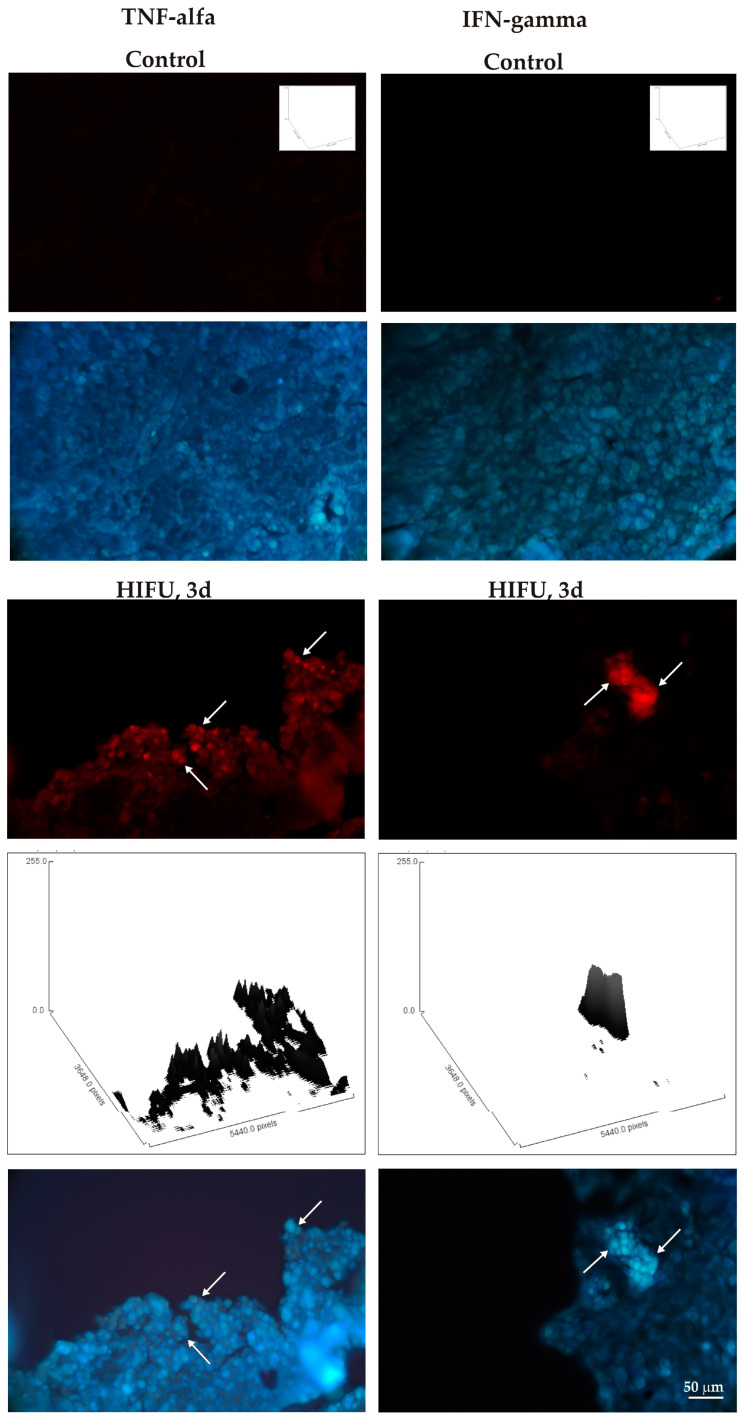
Immunofluorescence signals for TNF-α (red, (**left panel**)) and IFN-γ (**right panel**) in a HIFU-treated tumor (3 days after sonication), compared to those in a HIFU-untreated tumor. White arrows point at immunopositive cells. Bisbenzimide (blue) counterstains the cell nuclei. Histograms show the intensity of immunolabeling for TNF-α and IFN-γ per microscopic field of view. The intensity of immunoreactivity is presented as the height of the surface plot profiles.

In the next step, the expression of pro-inflammatory cytokines was studied in the control and HIFU-treated tumors. Three days after sonication, induction of immunoreactivity for pro-inflammatory cytokines was detected for IL-1α and IL-1β (Figure 11) and TNF-α and INF-γ; this was not observed in untreated tumor tissue (Figure 12).

To sum up, in the regions subjected to sonication, all cancer cells in the field of vision were destroyed. No differences were observed between the damage visible in cells at the margin of healthy tissues surrounding the tumor and the damage visible in cancer cells.

## 4. Discussion

The incidence rate of breast cancer among women is now approximately 10 per cent of all new malignancies [26], but the cancer is still one of the most deadly cancers in women [2]. Thanks to medical developments, breast cancer is often diagnosed at an early stage, when the prognosis is much better and allows for conservative therapy. Typical therapeutic management is based on mastectomy or breast-conserving surgery, chemotherapy, radiotherapy, and hormonal therapy [9]. The above techniques are either invasive or have serious side effects. Therefore, new minimally invasive therapeutic techniques are constantly being sought. These include minimally invasive or non-invasive ablation techniques.

The recent data indicate that the ablation technique for the treatment of breast cancer seems quite promising, but still requires detailed research and the development of new therapeutic devices and procedures that can be approved as effective and safe [25,27]. One of the ways to ablate a tumor located deep under the skin is to expose it to a pulsed HIFU beam with appropriate acoustic properties (frequency, intensity at the focus, and duty factor), beam movement trajectory, time intervals between consecutive single sonications, and exposure time. HIFU treatment is a promising transdermal technique due to its non-invasiveness (no surgery is needed), minimal undesirable side effects (skin and subcutaneous tissue burns, as noted by Fleming [28]), lack of ionizing radiation, possibility of repeating the treatment, and faster recovery, which is crucial for the safety of a therapy. 

To minimize unwanted side effects and destroy only tumor tissue with a predetermined healthy-tissue margin, the procedure is performed under imaging guidance. MRI or USI guidance is usually used with such devices, but the MRI-guided devices available on the market are very expensive. Although commercial therapeutic devices have been developed for humans [29,30], HIFU therapy can currently be used as an alternative to surgery only in a limited number of cases. Requirements include, but are not limited to, an early stage of tumor development, and a tumor size of up to 2 cm [31,32]. The long-term cure rates of clinical trials employing HIFU are often not clearly reported [33], but there is a clear need for improved treatment regimens. Development of new procedures to reduce treatment duration and complications (skin burns, pain, edema, hyperpigmentation, or hemorrhages) may help in solving this problem [33,34]). 

The automated ultrasound imaging-guided HIFU ablation device for thermal destruction of solid tumors in small animals developed at the IFTR and described here has proven to be an effective tool for this purpose, while minimizing damage to healthy tissues surrounding the tumor. The proposed ablation plan (Figure 2), which consists of shortening the time intervals between repeated single sonications, allows researchers to solve one of the major problems of HIFU therapy by significantly shortening the duration of the ablation procedure, and thus increasing its safety and reducing its costs. The appropriate acoustic parameters of the single pulsed HIFU beam and exposure time used in this study were determined in our previous experiments [23,24]. The time intervals between consecutive sonications were reduced from 120 s (determined by the temperature relaxation of the sonicated tissue) to 20 s. The choice of ultrasound imaging in our device to guide the focus of the HIFU beam to the targeted volume inside the tumor was dictated by economic and accessibility-based reasons.

Our results confirm the data of other researchers [6,7,9] and demonstrate the effectiveness of our HIFU ablation device in damaging breast cancer cells. They also show that HIFU ablation causes cell damage and degeneration and cell death by coagulative necrosis [18,35]. Gianfelice and co-authors demonstrated the presence of multiple hemorrhages and extravasations in HIFU-treated areas [36]. Merckel and colleagues detected massive leakages of red blood cells from damaged vessels after HIFU treatment [37]. Our research proves that HIFU therapy destroys cancer cells and blood vessels penetrating the tumor. Similar results have been obtained by others [18]. It is known that all tissue destruction techniques based on thermal effects result in the destruction of blood vessels in the area of the damaging agent [18]. In further studies, we plan to change the acoustic parameters of the device to achieve even greater destruction of blood vessels in the HIFU-treated tumor area.

An innovative solution in our research was the choice of an ablation procedure with a shortened duration, which proved that shortening the time intervals between single sonications did not increase the size of the necrotic lesion beyond the planned treated volume. Another benefit is the confirmation of the performance of a self-built low-cost device that is on a par with expensive commercial devices. 

A limitation of the ultrasound imaging-guided HIFU technique is that it is mostly suitable for thermal destruction of solid tumors with clearly defined, smooth contours on B-mode ultrasound images. This allows precise determination of the location and size of the tumor and an accurate determination of the tissue bulk to be ablated (including an appropriate margin of healthy tissues).

A common major drawback of using ablative techniques (including HIFU) for breast cancer treatment is that if a part of the tumor is skipped during the treatment procedure, such as in, e.g., cases due to patient movement, the therapy fails [27,33,38]. Therefore, it is recommended that the patient be immobilized and that the tumor be ablated along with a 2 cm margin of healthy tissue(s) [27]. Notably, a prominent tumor regrowth was visible within 3 days of sonication in the rats with some minor portion of the tumor left HIFU-untreated. This limitation also classifies the HIFU technique as more for the treatment of solid benign tumors, ruling out the use of the HIFU ablation technique to destroy irregularly shaped malignant tumors.

We also observed a decrease in the VEGF immunoreactivity within the treated tumor. This finding is consistent with previous studies that highlight the ability of HIFU to disrupt VEGF signaling pathways, possibly by inducing thermal stress and mechanical disturbances within the tumor [39,40].

The downregulation of VEGF immunoreactivity is a critical observation, as this phenomenon directly impacts tumor angiogenesis. VEGF is a potent pro-angiogenic factor that promotes the proliferation and migration of endothelial cells, leading to the formation of new blood vessels that supply nutrients and oxygen to the tumor. By lowering the level of VEGF in cancer cells, HIFU may impede the angiogenic switch that sustains tumor growth and metastasis [41].

Our findings provide compelling evidence that the proposed HIFU ablation procedure leads to extensive tumor necrosis. Histological analysis of treated tumor specimens revealed coagulative necrosis within the target region, characterized by cellular shrinkage, loss of nuclear staining, and tissue structure disruption. These histopathological changes are indicative of irreversible cell damage caused by the thermal and mechanical effects of HIFU, a finding consistent with previous studies [9,33].

Furthermore, our results underscore the role of HIFU in stimulating an inflammatory response within the treated tumor microenvironment. This is supported by the pronounced infiltration of immune cells, mainly neutrophils and macrophages, observed in tumors after HIFU treatment [42,43]. The influx of immune cells is indicative of an acute inflammatory response triggered by HIFU-induced tissue injury. This response is known to play a pivotal role in the clearance of necrotic debris and the initiation of an adaptive immune response against tumor antigens [34,38,44,45]. The pro-inflammatory cytokine profile in our study revealed a significant upregulation of key cytokines such as IL-1α, IL-1β, IFN-γ, and TNF-α following HIFU treatment. These cytokines are known to promote inflammation and recruit immune cells to the site of injury, further supporting the induction of an inflammatory milieu within the treated tumor [33,35,43,46]. The phenomena described above ultimately lead to rapid tumor destruction without causing excessive unwanted changes in the heathy tissue(s) surrounding the tumor.

In this project we constructed a custom, automated, ultrasound imaging-guided HIFU ablation device. Our device is low-cost, compact and portable, and the sonication protocol developed has significant benefits: it reduces the treatment duration and is less burdensome for the treated animal, which benefits its health parameters. The major novelty of our project is the development and verification of the effectiveness and safety of this novel sonication protocol. Treatment duration is one of the key factors in HIFU therapy, because the treatment requires the patient’s immobility. The majority of relevant studies do not provide an exact time of exposure to a single HIFU beam or the intervals between them during breast cancer therapy. Usually, the duration of the entire procedure is given, a value which varies between 35 min and (even) 145 min [9,36,37,47]. Therefore, the protocol we have developed, which allows for such a significant reduction in the duration of the ablation procedure, is of great benefit.

The results of our research may be useful not only for the treatment of breast cancer, but also for the treatment of other cancers, e.g., benign tumors such as uterine fibroids. To avoid the overheating of healthy tissue surrounding the tumor, an appropriate time interval must be maintained between successive sonications to allow the tumor to cool (in the conventional procedure, it takes 60–90 s), which significantly prolongs the duration of therapy. For example, in the case of a uterine fibroid, the cooling time can constitute more than 80% of the duration of ablation, which usually takes several hours (6–8 h) [48]. A whole-prostate HIFU ablation procedure, in a single session, requires a few hours (4–6 h) [49].

## 5. Conclusions

The results of this study demonstrated that our low-cost, compact, automated, ultrasound imaging-guided HIFU ablation device can be an effective tool for preclinical studies and the noninvasive transcutaneous treatment of solid tumors in small animals. The proposed reduction of the time interval between consecutive sonications allows significant shortening of the duration of the ablation procedure for the entire tumor volume with a given margin without damaging the surrounding healthy tissues. This also reduces the cost of the procedure and allows more procedures to be performed. In summary, the results of this preclinical study seem promising for the possible further development and refinement of the HIFU technique for the human oncology clinic.

## Data Availability

The data presented in this study are available in this article.
